# Supplementary material for: Changes in anthropometric indices, lifestyle patterns, and mental stress with ramadan intermittent fasting among healthy students: A prospective cohort study
Source: PLoS One. 2026 Apr 22;21(4):e0345940. doi: 10.1371/journal.pone.0345940 (PMC13102226; doi:10.1371/journal.pone.0345940)
Supplement: Attachment — Submitted filename: Reviwer_comments.docx [file pone.0345940.s002.docx]

**May 2, 2025**

**Dear Editor,**

It is a pleasure to resubmit the revised Manuscript Number: PONE-D-25-10924, titled "Changes in Anthropometric Indices, Lifestyle Patterns, and Mental Stress with Ramadan Intermittent Fasting among Healthy Students: A Prospective Cohort Study" for consideration as an article in PLOS ONE. We found the comments provided by the reviewers to be constructive and have improved the manuscript. We believe the manuscript is currently more suitable for publication in its revised format.

**Reviewer #1:**

**Title and text:**

‘Anthropometric Indices’ seems more appropriate than ‘obesity indices’, as well as ‘Lifestyle pattern/behaviour’ than ‘lifestyle medicine’ and ‘a prospective cohort study’ than ‘a competent cohort study’. Please re-write the title.

Response: I have changed it to “Changes in Anthropometric Indices, Lifestyle Patterns, and Mental Stress with Ramadan Intermittent Fasting among Healthy Students: A Prospective Cohort Study”

**Abstract:**

- AVI and BAI were not calculated in the present study. They figure in the abstract and the method section.

Response: We would like to clarify that the Abdominal Volume Index (AVI) was not calculated in the present study. However, the Body Adiposity Index (BAI) was indeed calculated and included in our analysis. We apologize for any confusion caused by the initial abstract and method section.

- Avoid the use of ‘never been studied’

Response: I have changed it to ‘as these have yet to be thoroughly investigated.’

- The number of participants (150; 153) and their age (18-40; 18-45), gender (100/ 95 female; and function (students; students and university employees) are different in the abstract, methods, and results sections

Response: Thank you for pointing out the inconsistencies in the number of participants, their age, gender distribution, and function across the abstract, methods, and results sections. We have reviewed the manuscript and made the necessary corrections to ensure consistency. The correct details are as follows:

Number of participants: 150

Age range: 18-40

Gender distribution: 92 females and 58 males

- Some units are lacking (Kg).

Response: We have reviewed the document and added the necessary units (Kg) where appropriate to ensure clarity and precision.

- Line 47: please reformulate the sentence.

Response: I have change it to “There are various methods for weight loss, such as physical activity, fasting, specific diets, medication, and hormonal treatments. Among these, intermittent fasting is particularly effective for losing weight.

- It is unclear if the data collected during the second visit concerned RIF or the week after RIF. Please clarify and correct accordingly in the text and tables.

Response: We would like to clarify that the data collected during the second visit concerned the period immediately after Ramadan Intermittent Fasting (RIF). We have corrected the text and tables accordingly to reflect this.

**Keywords**

- intermittent fasting and anthropometry or waist circumference (rather than obesity) can be added.

Response: We agree that "intermittent fasting and anthropometry" or "waist circumference" are more appropriate keywords than "obesity." We have updated the keywords accordingly.

**Introduction**

- Line 74: some repetition

Response: We have reviewed the manuscript and revised the text to eliminate the redundancy. The updated version is now clearer and more concise.

**Methods**

- Conditions of Ramadan 2024: please indicate the temperature and the exact duration of fasting.

Response: We would like to clarify that this prospective cohort study was conducted during Ramadan from March to April 2024 (Shaban-1445 to Ramadan-1445). During this period, the average temperature in Amman, Jordan ranged from 14°C to 25°C. The exact duration of fasting each day was approximately 13 to 14 hours. We have added these details to the relevant paragraph in the manuscript to ensure clarity.

- Please indicate the population’s life conditions: university housing?

Response: We would like to clarify that the participants in this study were university students and employees residing in university housing and private accommodations. This detail has been added to the relevant sections of the manuscript to ensure clarity.

- -101-102: sentence without a verb” The fasting period for premenopausal female participants from 23 to 25 days, whereas the fasting period for the men from 28 to 30 days from 13 to 14 hr.”

Response: We have revised the sentence for clarity. The corrected sentence now reads: "The fasting period lasted from 23 to 25 days for premenopausal female participants, whereas for men, it lasted from 28 to 30 days, with each day involving 13 to 14 hours of fasting."

- -105 and 110: repetition

Response: We have reviewed the manuscript and revised the text to eliminate the redundancy. The updated version is now clearer and more concise.

- Figure 1 is lacking

Response: We have reviewed the manuscript and included Figure 1 to ensure completeness. The figure provides a visual representation of the study design and data collection timeline, I have added it to the manuscript

- Inclusion criteria: add ‘patients willing to fast during Ramadan 2024’.

Response: The inclusion criteria were healthy individuals who did not complain of any chronic diseases or mental illnesses and were willing to fast during Ramadan 2024.

- Please describe the term ‘professional staff’

Response: We would like to clarify that "professional staff" refers to trained and qualified personnel, including dietitians, clinical nutritionists, and clinical pharmacists, who conducted the questionnaires and face-to-face interviews. These individuals possess the necessary expertise to accurately gather demographic data, lifestyle information, and medical history from the participants

I have change it to (Using questionnaires and face-to-face interviews done by trained and qualified personnel, including dietitians, clinical nutritionistsdemographic data including age, sex, marital status, education level, lifestyle data (physical activity and sleep hours),

- Please indicate how many times dietary intake was evaluated (line 118).

Response: Dietary habits were assessed by a trained dietician at two points: the initial visit and the follow-up immediately after one week of RIF**.** Information on dietary intake was obtained using a validated short food frequency questionnaire containing 22 items to evaluate fat, sugar, fruit, vegetable, and water intake.

- The technique of hip circumference measurement is lacking (lines 136-137).

Response: I have change it to “WC and HC were measured while standing using the RIEDER Body Measure 60/150 cm (Inct. Bonus Kit, REIDHK®, China). Waist circumference was measured on the horizontal plane midway between the lowest rib and the iliac crest. Hip circumference was measured by wrapping the measuring tape around the widest part of the hips, ensuring it was parallel to the floor”

- The formulas are unclear, please re-write and use ‘x’ instead of ‘*’.

Response: I have reviewed all formulas and ensured their accuracy. The formulas are now presented using 'x' instead of '*'.

- CI and WHtR indices were not defined.

Response: We have addressed the concerns regarding the definitions of the Cardiac Index (CI) and Waist-to-Height Ratio (WHtR). We have reviewed and ensured that these definitions are consistently applied throughout the paper.

- -please use only one abbreviation: IRF or RF.

Response: we have standardized the abbreviation for Ramadan fasting (RF) throughout the text to maintain consistency.

**Results**

- The number of fasted days in the subjects was not mentioned.

Response: Thank you for your valuable feedback. We have addressed the concern regarding the number of fasted days. This information has been included in the methods section of the paper.

- Please verify the numbering of the paragraphs and the tables.

Response: We have verified the numbering of the paragraphs and tables to ensure accuracy and consistency throughout the document.

- Line 165: JD?

Response: we have clarified that "JD" refers to Jordanian Dinar and have converted the amount to Dollar for better understanding.

- Table 1: Please verify, the age, and sex of the population. The total for each parameter should be 100%. Income (Equivalent in dollars).

Response: We have verified the age and sex distribution of the population, ensuring that the total for each parameter sums to 100%. We apologize for any oversight and appreciate your attention to detail.

- Table 3 (please correct the number): perceived stress level: it should rather be expressed by the number of subjects for each category.

Response: Thank you for your valuable feedback. We have corrected the numbering of Table 3. Additionally, we have revised the table to express the perceived stress level by the number of subjects for each category.

- Table 4: data should be expressed as mean and SD (if normally distributed). Daily calories intake was not calculated. It seems important to discuss weight loss.

Response: We have ensured that the data is expressed as mean and standard deviation (SD) where normally distributed. Regarding daily calorie intake, we acknowledge that it was not calculated in the current study. This limitation has been added to the manuscript.

- The sentence in lines 182-183 can be displaced to line 176 with table 3 data and ‘however’ should be deleted.

Response: We have addressed the concern regarding the placement of the sentence in lines 182-183. The sentence has been moved to line 176 along with the data from Table 3, and the word "however" has been deleted for clarity.

**Discussion**

- Lines 187-190: please reformulate as no analysis of the changes in anthropometric indices and lifestyle patterns was performed

Response: I have change it to “The current study aimed to examine the impact of Ramadan fasting (RF) on obesity indices, daily habits, nutrient intake, and mental stress in health students. However, no analysis of the changes in anthropometric indices and lifestyle patterns was performed. This contributes to a deeper understanding of the potential health implications of RF in young adults”

- -Line 195: Please correct: there was a significant decrease in BMI.

Response: I have rewrite it to be "The reduction in BMI is significant, but not with other anthropometric indices related to obesity. This is likely due to the significant increase in consumption of pastries, desserts, and processed meat, which affects most obesity indices (i.e., BMI, WC, ABSI, CI, and WWI."

- -Lines 200-207: useless here as these data were not studied in the present study

Response: These data were not studied in the present study, so they have been deleted.

- Lines 213; and 214: please use the past tense

Response: The revised sentence using the past tense: "Khan et al. (2017) found that there was no significant change in the anthropometric parameters of the thirty-five medical students in Pakistan; these parameters included weight, BMI, WHtR, BAI, and VAI [34]."

- Subjects lost 6 Kg in weight: this important weight loss should be discussed (IRF, living conditions, dehydration…).

Response: I've added a paragraph discussing the significant weight loss observed in subjects, highlighting factors such as Intermittent Ramadan Fasting (IRF), living conditions, and dehydration. This addition provides a comprehensive understanding of the mechanisms behind the weight reduction

- The changes in the different anthropometric indices were not discussed; what does it mean? what is the importance of the calculation of these indices?

Response: I have added this paragraph ‘The change in WC was addressed partially as a result of reduced total body water and fat mass. Other recent studies also showed the same results regarding the effect of RIF on weight loss and other anthropometric [2,5,9,10]. On the other hand, Das et al. (2019) conducted a study on a population of Indian Muslims after Ramadan. The results showed an increase in body weight and BMI after one month of Ramadan [11]. Additionally, there is a lack of studies examining the effects of Ramadan fasting on Conicity Index (CI), Abdominal Volume Index (AVI), and Body Adiposity Index (BAI). The current study found that there was no significant different among this parameter The mechanism behind the lack of significant differences in CI, AVI, and BAI during Ramadan fasting could be attributed to the body's metabolic adaptations[12]. During fasting, the body shifts from using glycogen stores to burning fat for energy, leading to overall weight and fat mass reduction[5]. However, these changes may not significantly impact specific adiposity indices like CI, AVI, and BAI, which are more sensitive to long-term body composition changes rather than short-term weight fluctuations’

- -Line 271: the sample size was considered adequate? How?

Response: Sorry, I have changed it to: 'Furthermore, the study's sample size was relatively small, which could be expanded in future research to enhance the robustness of the findings.

- Line 273: which variables do the authors mean?

Response: I have mentioned it in the paragraph

**Conclusion**:

Improvements in dietary habits? Sleep duration? Please re-write the conclusion.

Response: I have rewrite it to be “The RIF positively impacts lifestyle parameters, body weight, and stress levels among university students and employees. Significant improvements were observed in dietary habits and sleep duration, contributing to overall well-being. Obesity indices, including reductions in body weight, BMI, and BRI, showed notable improvements. Additionally, stress levels decreased significantly during RIF, highlighting its potential benefits for mental health. These findings suggest that RIF can be an effective strategy for enhancing both physical and psychological health.

**References:**

- There are too many references (52).

Response: I have made it 48

**Reviewer #2**

**Abstract:**

- • "The association between Ramadan Intermittent Fasting (RIF) and metabolic profiles has been studies." should be "has been studied."
- • The sentence, "RIF fasting positive body weight, obesity indices, and stress levels among health students," is unclear. It seems incomplete and should be rephrased for clarity.

Response: Thank you for your feedback. I have made the necessary revisions:

- "The association between Ramadan Intermittent Fasting (RIF) and metabolic profiles has been studied."
- "RIF fasting positively impacts body weight, obesity indices, and stress levels among healthy students. These findings suggest that RIF can improve health outcomes by promoting better dietary habits, enhancing sleep duration, and reducing stress."
- The abstract mentions significant reductions in various indices but does not include full p-values for all reported results, which would enhance the clarity and statistical rigor of the findings

Response: I have added the p value p>0.05.

- The results about changes in obesity indices are somewhat disjointed. The abstract lists significant changes in BMI, WHtR, and BRI, but other indices are not clearly presented. A more structured presentation of results would improve readability.

Response: I rewrite the abstract to make it clearer

‘The association between Ramadan Intermittent Fasting (RIF) and metabolic profiles has been studied. However, there remains a significant gap in the literature regarding the changes in obesity indices during RIF, as these have never been thoroughly investigated. Additionally, the role of lifestyle medicine in conjunction with RIF has not been thoroughly explored. This paper aims to address these gaps by examining the impact of RIF on obesity anthropometric indices, lifestyle patterns, and mental stress. This prospective cohort study involved 150 university students aged 18-40 years. Data was collected using questionnaires and face-to-face interviews conducted one week before and immediately after one week of RIF. Body weight and height were measured to calculate body mass index (BMI) and other obesity indices, including Conicity Index (CI), Abdominal Volume Index (AVI), Body Adiposity Index (BAI), Weight-Adjusted-Waist Index (WWI), and Body Roundness Index (BRI). The Waist-to-Height Ratio (WHtR) showed a significant decrease from 0.481±0.11 to 0.45±0.10, with reductions in body weight (kg) (62.63±1.2 vs. 69.25±1.18, p<0.05). A significant change in BRI before and after fasting (1.84±0.37 vs. 1.06±0.17, p<0.05) was observed. However, there was no significant difference in other obesity indices (p>0.05) such as CI, WWI, and BAI. Significant changes were observed in lifestyle parameters after RIF, including decreased sleep hours (6 ± 0.263 vs. 8 ± 0.581, p<0.05) and reduced number of meals after fasting (1 ± 0.135 vs. 3 ± 1.242, p<0.05). Stress levels significantly decreased after RIF (p<0.05). RIF fasting positively impacts body weight, obesity indices, and stress levels among healthy students. These findings suggest that RIF can improve health outcomes by promoting better dietary habits, enhancing sleep duration, and reducing stress, leading to significant reductions in body weight, BMI, and BRI.

**Introduction**

- The flow between topics (from obesity to lifestyle medicine to fasting) is somewhat abrupt. For example, the transition from discussing obesity indices to lifestyle medicine could be smoother, providing a clearer link between these areas and RIF.

Response: I have carefully reviewed your comments on introduction and made the necessary revisions to improve the flow and clarity of the manuscript.

- "Lifestyle medicine is an emerging discipline that emphasizes the prevention and management of diseases linked to unhealthy lifestyle choices." Add reference like this study to this sentence: DOI: 10.1093/nutrit/nuad096 and DOI: 10.2174/1871530321666210316103403

Response: I have add reference to the sentence “Lifestyle medicine is an emerging discipline that emphasizes the prevention and management of diseases linked to unhealthy lifestyle choices[5].”

- The sentence "Fasting is considered to be a healthy practice, recent studies have shown a positive association between fasting and longevity" could be more precise. It would be beneficial to specify whether the longevity mentioned refers to Ramadan fasting specifically or fasting in general.

Response : I have rewrite it to make it clear “Fasting is considered a healthy practice, with recent studies showing a positive association between various forms of fasting, including intermittent fasting and Ramadan fasting, and longevity Fasting is considered to be a healthy practice, recent studies have shown a positive association between fasting and longevity [10,11]

**Method**

- While the sample size of 150 participants is mentioned, there is no mention of a power analysis to justify this sample size. A power analysis would help assess whether this sample size is sufficient to detect statistically significant differences and strengthen the study’s statistical reliability

Response: this is the revised paragraph with the power analysis included in the Study Participants section: “This prospective cohort study was conducted on 150 university students (58 males and 100 92 females), aged 18-40 years old. Informed consent had been obtained. The inclusion criteria were healthy individuals who did not complain of any chronic diseases or mental illnesses and were willing to fast during Ramadan 2024The inclusion criteria were healthy and did not complain of any chronic diseases or mental illnesses. A calculation of the sample size was conducted to ascertain the necessary number of participants. Drawing from earlier quasi-experimental studies, it was determined that at least 70 participants were required to attain 80% power with a p-value of 0.05 in order to identify a significant difference of 1 kg in BMI before and after fasting. This estimation was based on previous research indicating an anticipated effect size of 0.20[21].”.

- The description of data collection (questionnaires, interviews) could include more detail on how these tools were administered. Were the interviews standardized? Were they conducted in person or online? Providing more specifics here would increase transparency and replicability

Response: I have revised the description of the data collection methods to include more detail on how these tools were administered

- It would be useful to mention whether any control variables were considered, especially for lifestyle factors like diet, physical activity, or mental health status before the study began. Acknowledging or controlling for such factors is important in determining whether changes in outcomes are truly attributable to Ramadan fasting.

Response: To ensure that changes in outcomes were truly attributable to Ramadan fasting, several control variables were considered. These included baseline measurements of dietary habits, physical activity levels, and mental health status before the study began. By acknowledging and controlling for these factors, we aimed to isolate the effects of Ramadan fasting on the observed changes in obesity indices, lifestyle patterns, and mental stress.

- It would be useful to mention whether any control variables were considered, especially for lifestyle factors like diet, physical activity, or mental health status before the study began. Acknowledging or controlling for such factors is important in determining whether changes in outcomes are truly attributable to Ramadan fasting.

Response: this is the revised section with the control variables integrated into the data collection description “To ensure that changes in outcomes were truly attributable to Ramadan fasting, baseline measurements of dietary habits, physical activity levels, and mental health status were taken before the study began. These control variables were considered in the analysis to isolate the effects of Ramadan fasting on the observed changes in obesity indices, lifestyle patterns, and mental stress”.

**Result**

- While paired t-tests are used, it would be helpful to also report effect sizes (such as Cohen’s d) to provide a sense of the practical significance of the findings. This would aid readers in understanding the magnitude of the changes, not just their statistical significance.

Response: Thank you for your valuable suggestion regarding the inclusion of effect sizes to complement the paired t-tests. We have now added Cohen's d effect sizes to the result tables to provide a clearer understanding of the practical significance of our findings. This addition helps to illustrate the magnitude of the changes observed, not just their statistical significance.

- Updated Tables:

We have updated Tables 2 and 3 to include Cohen's d values alongside the p-values. These effect sizes offer a more comprehensive view of the impact of Ramadan Intermittent Fasting (RIF) on various obesity indices and lifestyle parameters.

- The text notes that there was "no significant difference in other obesity indices" but doesn’t explain why certain indices like BMI, WHtR, and BRI showed significant changes while others did not. A more thorough analysis of why some measures responded to fasting and others did not would be valuable for interpretation.

Response: Thank you for your insightful comments regarding the observed changes in obesity indices following Ramadan Intermittent Fasting (RIF). We appreciate your suggestion to provide a more thorough analysis of why certain measures, such as BMI, WHtR, and BRI, showed significant changes while others did not.

Analysis:

The significant changes in BMI, WHtR, and BRI can be attributed to the direct impact of fasting on body weight and fat distribution. Ramadan fasting involves prolonged periods of abstinence from food and drink, leading to a caloric deficit and subsequent weight loss. This weight loss is reflected in the reduction of BMI, which is a measure of body weight relative to height. Similarly, WHtR and BRI are sensitive to changes in abdominal fat and overall body shape, which are directly influenced by the reduction in caloric intake and changes in dietary habits during fasting.

On the other hand, indices such as CI, WWI, and BAI did not show significant changes. This lack of significant difference can be explained by several factors:

1.	Sensitivity to Short-Term Changes: CI, WWI, and BAI are more sensitive to long-term changes in body composition rather than short-term fluctuations. Ramadan fasting, while effective in reducing overall body weight, may not induce significant changes in these indices within the short duration of the fasting period.

2.	Dietary Composition: The increase in consumption of high-calorie foods, pastries, and desserts during Ramadan, as noted in our study, may counteract the potential benefits of fasting on these specific indices. While overall caloric intake is reduced, the quality of the diet may not support significant changes in CI, WWI, and BAI.

3.	Metabolic Adaptations: The body's metabolic adaptations during fasting, such as shifts in energy utilization from glycogen to fat stores, may not significantly impact these indices. CI, WWI, and BAI are more reflective of chronic changes in fat distribution and metabolic health, which require longer periods to manifest.

4.	Hydration Status: Changes in hydration status during fasting can affect measurements of body composition. Indices like CI and BAI may be influenced by fluctuations in body water content, which can vary significantly during fasting periods.

**Discussion**

- The discussion implies a causal relationship between Ramadan fasting and changes in health outcomes. However, the study’s design is observational, which means it can only show associations, not causality. The authors should be cautious in their language and clarify that the findings represent associations, not causal effects.

Response : Thank you for your insightful feedback. We acknowledge the importance of accurately representing the nature of our study. While our study design is observational, it is specifically a prospective cohort study, which involves measuring the same sample at two different time points. I have revised the manuscript to clarify that our findings represent associations observed within a prospective cohort study, rather than implying causality. This approach allows us to observe changes over time within the same group of participants, providing a stronger basis for identifying associations between Ramadan fasting and health outcomes. Thank you for your valuable input, which will help improve the clarity and accuracy of our manuscript.

- While the manuscript compares its results to previous studies, it would be useful to better reconcile the differences in findings with potential methodological differences between studies. For example, what are the reasons for discrepancies in findings about stress and weight gain/loss? Are these linked to differences in study design, fasting durations, or cultural dietary habits?

Response : thank you for this comment I have added this paragraph “Fasting durations during Ramadan vary from 12 to 20 hours based on location and time of year, affecting physiological and psychological responses[39]. Longer fasting periods can alter metabolic responses, hydration, and energy balance, contributing to study variations [16]. Additionally, cultural dietary habits during Ramadan differ, with some regions consuming more sweets and fried foods, while others focus on balanced meals[17]. These dietary differences impact weight changes, metabolic health, and stress levels, leading to discrepancies in study outcomes.

- Although the authors briefly mention the need for larger sample sizes and more homogeneous groups, the discussion could benefit from more specific suggestions for future studies. For instance, longitudinal studies following participants over a longer period would help assess the long-term effects of Ramadan fasting on health outcomes.

Response: Thank you for your valuable feedback. We appreciate your suggestion to provide more specific recommendations for future studies. We have revised the discussion section
