# Supplementary material for: Changes in anthropometric indices, lifestyle patterns, and mental stress with ramadan intermittent fasting among healthy students: A prospective cohort study
Source: PLoS One. 2026 Apr 22;21(4):e0345940. doi: 10.1371/journal.pone.0345940 (PMC13102226; doi:10.1371/journal.pone.0345940)
Supplement: Attachment — Submitted filename: Reviwer_comments_auresp_2.docx [file pone.0345940.s003.docx]

**February 19, 2026**

**Dear Editor,**

It is a pleasure to resubmit the revised Manuscript Number: PONE-D-25-10924, titled "Changes in Anthropometric Indices, Lifestyle Patterns, and Mental Stress with Ramadan Intermittent Fasting among Healthy Students: A Prospective Cohort Study" for consideration as an article in PLOS ONE. We found the comments provided by the reviewers to be constructive and have improved the manuscript. We believe the manuscript is currently more suitable for publication in its revised format.

**Reviewer**

**Comment**: I appreciate your manuscript as it explores a relatively unique and less researched area. While I commend the overall structure and content of the manuscript, I have noted several areas where enhancements could be made.
• The document is slightly bulky and has too many references

**Response:** We have reduced the number of references by removing repetitive and less directly relevant citations. Redundant explanations were shortened, and sections were streamlined to improve conciseness without compromising scientific integrity.

**Comment** Your document needs a through grammatical and editorial revision

**Response:** The manuscript has undergone comprehensive grammatical and language editing. Sentences were restructured for clarity, coherence, and academic tone throughout the manuscript.

### Abstract

### Comment: On line 24 ‘anthropometric indices related to obesity during RIF…’ since your study population is not defined by the BMI or whether they are obese or not replace the OBESITY with more general term like WEIGHT or other suitable one as all your study participants are not obese

### ****Response**:** The term “obesity” has been replaced with “body weight” to better reflect the characteristics of our study population.

### Comment: Also, on line 24 you have used, these have never been thoroughly investigated’ which is an absolute language please replace with more qualified statement to convey the gap like ‘these have not been extensively investigated’

### ****Response**: done I have changed it**

### • Comment: On line 36 better to remove (p>0.05)

### ****Response**: done I have changed it**

**Introduction**

**Comment** 1. You did not pick the main topic of your manuscript, RIF until the third paragraph I would recommend you to pick earlier (in the first or 2nd paragraph) after your brief opening so that the reader quickly gets the main point of interest

**Response:** The introduction has been reorganized. Ramadan Intermittent Fasting (RIF) is now introduced in the second paragraph to clearly establish the main focus of the study earlier.

**Comment** 2. Line 72 ‘…Muslims fast daily from m dawn to sunset’ writing errors are plenty in your document, please revise thoroughly

**Response:** All typographical and grammatical errors have been corrected after thorough revision.

**Methodology**

**Comment**: 1. Line 89 ‘...during Ramadan from March to April 2024 (Shaban-1445 to Ramadan-1445) these two time frames are conflicting, may you please remove DURING RAMADAN or change to during the months of Shaban and Ramadan

**Response:** Revised to: “This prospective cohort study was conducted during the months of Shaban and Ramadan (March–April 2024).”
**Comment** :2. Line 90-91 and line 135 ‘... on obesity indices...’ as your study is not confined to only using obesity indices (Conicity Index (CI), Abdominal Volume Index (AVI), Body Adiposity Index (BAI), Weight-Adjusted-Waist Index (WWI), and Body Roundness Index (BRI).) which are often designed for fat distribution estimation, rephrase and add the other anthropometric indices (BMI etc) by saying ( .. Obesity and other anthropometric indices ...’ or you can use the more general term which is Anthropometric indices as all obesity indices are anthropometric indices, but not all anthropometric indices are obesity indices

**Response:** IN MANY STUDY THE USE OBESITY INDCES AS TERM FOR THESE VAERABLE

**Comment**. Line 103 regarding study participant ‘..This prospective cohort study was conducted on 150 university students (58 males and 92 females), was taking this male to female ratio a predetermined plaplan?herwise this is not expected here in methodology instead you describe the sociodemographic characteristics of your participants at the beginning of your result part

**Response:** The sex distribution has been removed from the methodology section and is now presented only in the Results section
4. Your methodology part needs the following considerations:
**Comment** Add at least one subtitle which addresses variables under which you put your dependent (effect of RIF on anthropometric indices...) and independent variables (socio economic characteristics, dietary, sleep and other factors you have assessed including anthropometric and obesity indices)

**Response: I add it in line 85**

**Comment** :line 88, instead of putting Study design alone, you would make (Study design, area and period)
• the other important missing section is Sampling technique or procedure which tells how you selected your study participants. Is it randomly or...?

**Response**: A new subsection titled “Study Participants and Sampling Procedure” has been added line 97 . We clarified that participants were recruited using a convenience sampling technique among eligible university students who intended to fast during Ramadan.
• you may also need to add operational terms section which defines your main operational terms like PSS

**Comment** for the readers and instead of defining those terms sparsely in the document, I would rather recommend you to combine them under definition of terms.

**Response:** **"Thank you for your recommendation. After careful consideration, I believe that defining the key terms contextually within the relevant sections ensures clarity and readability. Therefore, I prefer to retain the current structure rather than creating a separate section for operational definitions."**
**Comment** • In your analysis, in addition to p-value why don’t you add confidence interval(CI), as the confidence interval gives you a range within which you can be reasonably confident that the true mean difference lies
Result

**Response:** Thank you for this important methodological suggestion. We agree that reporting confidence intervals enhances the interpretability and statistical robustness of the findings. Accordingly, we have now calculated and added the 95% confidence intervals (95% CI) for the mean differences in the Results section and corresponding tables. This provides a clearer estimate of the precision and magnitude of the observed effects alongside the p-values and Cohen’s d effect sizes.

**Comment** 1. Line 174-175, you said ‘The demographics and 175 clinical information of the study participants are shown in Table 1’ but to avoid bulky document, just put (Table 1 or see Table 1) at the end of your first paragraph (line 174) and remove the long statement.

**Response:** Thank you for this valuable suggestion. We have revised the Results section accordingly. The separate sentence referring to Table 1 has been removed, and the table citation is now incorporated concisely at the end of the first paragraph as “(Table 1)” to improve clarity and reduce redundancy.

**Discussion**

**Comment** 1. Revise the first paragraph of your discussion as it has the following problems:
• You have started with some background and objectives of your study, instead focus on your result not objectives, aim or background information

**Response** The first paragraph has been completely rewritten. It now begins directly with a concise summary of the key findings (weight reduction, BMI and BRI changes, lifestyle and stress modifications) and integrates them cohesively.

**Comment** 2 The paragraph is fragmented and has poor coherence eg. Line 204-210

**Response: I have changed it**

**Comment** 2. There are contradicting statement about your study population in which you mentioned your study participants as University students but in discussion line 299-301, you said ‘This study has several limitations that should be acknowledged. Firstly, the heterogeneity of the participants, which included both university students and employees, may have influenced the results.’

**Response:** Thank you for identifying this inconsistency. The study population consisted exclusively of university students. The reference to “employees” has been removed from the Discussion and limitations section to ensure consistency throughout the manuscript.

**Comment** The abstract should be reorganised into clearly labelled sections such as Background, Methods, Result and Conclusion.

**Response: I have written the abstract as sections**
